# Tank-Binding Kinase 1 protects against MASH progression via mitochondrial quality control

**DOI:** 10.21203/rs.3.rs-7476559/v1

**Published:** 2025-10-09

**Authors:** Jin Young Huh, Sung-Min An, Jun Hee Jang, Jin Hyun Sung, Ji Won Myung, Yong Geun Jeon, Won Taek Lee, Jin Won Jeon, Kyung Min Yim, Jae-Ho Lee, Bichen Zhang, Jong Bae Seo, Seung Soon Im, Jae Bum Kim, Alan Saltiel

**Affiliations:** Sogang University; Sogang University; Sogang University; Sogang University; Sogang University; Seoul National University; Seoul National University; Sogang University; Seoul National University; Gyeongsang National University; University of California San Diego; Mokpo National Univ.; Keimyung University School of Medicine; Seoul National University; University of California San Diego

**Keywords:** Tank-binding kinase 1 (TBK1), Metabolic dysfunction-associated steatotic liver disease (MASLD), MASH, mitophagy, lysosome activity

## Abstract

Mitochondrial dysfunction is a critical driver of metabolic dysfunction–associated steatotic liver disease (MASLD) progression to steatohepatitis (MASH), yet the mechanisms governing mitochondrial quality control in hepatocytes remain poorly defined. Here, we identify TANK-binding kinase 1 (TBK1) as an essential regulator of hepatic mitophagy and lysosomal activity. Using TBK1-deficient hepatocytes and liver-specific TBK1 knockout (LTKO) mice, we show that TBK1 loss leads to the accumulation of depolarized, ROS-producing mitochondria due to impaired mitophagy flux, including defective lysosomal degradation. Mechanistically, TBK1 is required for p62 phosphorylation at Ser403 and partially modulates mTOR signaling to preserve lysosomal acidification. Therapeutic restoration of TBK1 expression via AAV8 delivery enhanced mitophagy, reduced mitochondrial burden, and ameliorated liver fibrosis. Notably, both human samples and murine steatohepatitis models exhibited a significant decline in TBK1 kinase activity. Collectively, these findings establish TBK1 as a critical guardian of mitochondrial and lysosomal homeostasis in MASH.

## INTRODUCTION

Metabolic dysfunction -associated steatotic liver disease (MASLD), previously known as nonalcoholic fatty liver disease (NAFLD), is a hepatic manifestation of metabolic syndrome and is closely correlated with obesity, one of the most significant risk factors for the disease^1^. The global rise in obesity has led to an increased prevalence of MASLD, making it one of the most common chronic liver diseases worldwide^2^. The interplay between excessive lipid accumulation in hepatocytes and mitochondrial dysfunction is central to the pathogenesis of MASLD, particularly as the disease progresses toward metabolic dysfunction-associated steatohepatitis (MASH)^3^. Mitochondria are critical for fatty acid oxidation and ATP production in the liver. However, in MASLD, the accumulation of lipids overwhelms mitochondrial capacity, leading to structural abnormalities such as fragmentation and swelling, as well as functional impairments like reduced oxidative phosphorylation and increased reactive oxygen species (ROS) production^3–6^. These mitochondrial defects contribute significantly to liver cell damage and the progression of MASLD to more severe stages, including MASH.

A key aspect of mitochondrial health is the maintenance of mitochondrial quality control mechanisms, which include mitochondrial biogenesis, fusion and fission, and mitophagy^7^. These processes are vital for maintaining mitochondrial function and preventing the accumulation of damaged organelles. Disruptions in these mechanisms are frequently observed in MASH, resulting in an imbalance between mitochondrial production and degradation, ultimately contributing to cellular dysfunction and disease progression^8^. Despite the recognized importance of mitochondrial dysfunction in MASH, the regulatory mechanisms underlying mitochondrial quality control remain poorly understood in the context of MASH.

Recent research has begun to elucidate the role of TANK-binding kinase 1 (TBK1), a serine/threonine kinase known for its involvement in multiple cellular processes, particularly autophagy^9^, innate immunity^10^, and energy metabolism^11–13^. Our previous study revealed that TBK1 plays a crucial role in hepatic lipid consumption via hepatic fatty acid oxidation^11^. The study demonstrated that liver (hepatocyte)-specific TBK1 knockout (LTKO) in mice led to reduced fatty acid oxidation via its scaffolding function, despite an increased number of mitochondria. This paradoxical increase in mitochondrial number suggested a complex regulatory role for TBK1 in mitochondrial quality control, and underscored the importance of further investigating TBK1’s role in mitochondrial regulation, particularly in the context of MASH progression. We demonstrate here that TBK1 is a key regulator of hepatic mitophagy and mitochondrial quality control that is dysregulated in MASH. By investigating the function of TBK1 in MASH pathogenesis, we aim to provide new insights into the complex role of TBK1 in the progression of MASH.

## MATERIALS AND METHODS

### Animals and Treatments

All mice were maintained on a C57BL/6J background. Liver-specific TBK1 knockout (LTKO) mice were generated by crossing TBK1 f/f (floxed) mice ^14^ with Albumin-Cre transgenic mice (Jackson Laboratory). TBK1 f/f mice were bred with TBK1 f/f with Cre/+ mice to generate TBK1 f/f (flox) and TBK1 f/f ^Cre/+^ (LTKO) littermates. Only male mice were used for all experiments. To induce diet-induced obesity and metabolic dysfunction, 8-week-old flox and LTKO mice were fed a 60% high-fat diet (HFD; Research Diets Inc., D12492) for 8 weeks. For fasting experiments, mice were subjected to an overnight fast (16 hours) before sample collection. For the MASLD mouse model, *ob/ob* mice were fed either a normal chow diet (NCD) or a high-fat, high-fructose, high-cholesterol (GAN) diet (Research Diets Inc., D09100310) for 3–4 weeks, starting at 8 weeks of age, to mimic simple steatotic liver and MASH liver, respectively. Age-matched *ob/+* mice were compared as lean controls. To overexpress human TBK1 *in vivo*, the cDNA sequence (NM_013254.3) was cloned into an AAV8 expression plasmid under the control of a CBh promoter (Vectorbuilder). For AAV infection experiments, AAV8-GFP or AAV8-human TBK1 (2 × 10^11^ genome copies per mouse) was injected into 8-week-old *ob/ob* mice. Mice were allowed 3 weeks for TBK1 overexpression, after which they were placed on the GAN diet for an additional 4 weeks before tissue and blood collection. Mice were housed in a specific pathogen-free (SPF) facility under a 12-hour light/dark cycle with ad libitum access to food and water, except during designated fasting periods. All experimental procedures were approved by the Institutional Animal Care and Use Committee (IACUC) at the University of California, San Diego, or at Seoul National University and were conducted in accordance with institutional and national ethical guidelines.

### Generation of TBK1 knockout HepG2 cells

RNA-guided targeting of human TBK1 in HepG2 cells was achieved through transfection of the Cas9 vector including guide RNA (gRNA). The gRNA was subcloned into Cas9 vector pSpCAS0(BB)-2A-Puro as described^15^. The 23 base pair genomic targeting sequence of the human TBK1 gRNA was 5’-AAAATGTTTACAGCTTCCAG-3’. HepG2 cells were transfected with Cas9 vector containing gRNA using Lipofectamine 3000 (Invitrogen), and selected using 3 μg/ml of puromycin for 2 days. Selected cells were seeded by single cell in 96-well plates for generation of monoclonal cell line. The used monoclonal TBK1-deleted HepG2 cell was confirmed by detecting endogenous TBK1 protein by immunoblot.

### Mouse primary hepatocyte isolation

Mouse primary hepatocytes were isolated from 8 to 12-week-old NCD-fed flox or LTKO male mice or C57BL6/J male mice as described previously ^11^. After 16 h fasting, mice were infused through the inferior vena cava with 25 ml of perfusion buffer (138 mM NaCl, 5.4 mM KCl, 0.6 mM NaH_2_PO_4_.H_2_O, 0.8 mM Na_2_HPO_4_, 10 mM HEPES, 4.2 mM NaHCO_3_, 0.5 mM EGTA, 5mM glucose, pH 7.4) for 3 min and followed by 25 ml of digestion buffer (40 μg/ml of Liberase TM (Roche), 138 mM NaCl, 5.4 mM KCl, 0.6 mM NaH_2_PO_4_.H_2_O, 0.8 mM Na_2_HPO_4_, 10 mM HEPES, 4.2 mM NaHCO_3_, 5 mM CaCl_2_, pH 7.4) for 3 min by using peristaltic pump. Liver tissues were washed with 25 ml of perfusion buffer for 3 min after digestion. Excised livers were minced and centrifuged at 50 × g for 1 min. Dead cells were excluded by removing the floating cells after centrifugation at 100 × g for 10 min in 36% percoll solution (Cytiva, GE17089101). Cells were resuspended with William’s medium E without glutamine (Life Technologies, 12551–032) supplemented with 10% of fetal bovine serum (FBS), GlutaMax (Life Technologies, 35050–061), and 1% penicillin/streptomycin to make 3×10^5^ cells/ml. 0.5 ml, 1 ml, 2 ml of cells were plated in 24-well, 12-well, and 6-well collagen-coated plates. After 4 h incubation for cell attachment, the media was replaced with fresh media. All experiments with primary hepatocytes were performed within 48 hours after isolation.

### Transmission Electron Microscopy for liver tissue

For mitochondrial morphology analysis for TBK1 deficient liver, 16-week-old flox and LTKO mice were used. The detailed procedure has been described previously ^11^. For mitochondrial morphology analysis for AAV8-TBK1 overexpression, liver tissues were sliced at 2 mm × 2 mm × 2 mm cube and put in fixative overnight, post-fixed in 1% osmium tetroxide in 0.1 M sodium cacodylate buffer for 1 h on ice, and stained en bloc with 0.5% uranyl acetate for overnight at 4°C. The stained tissues were dehydrated in ethanol (30–100%) and embedded with Spurr’s resin. Ultrathin (50–60 nm) sections were post-stained with uranyl acetate and lead stain. Samples were viewed using a JEOL JEM1010 (JEOL). For quantification, 48 images for AAV8-GFP group and 27 images AAV8-TBK1 overexpression group were analyzed by using Image J.

### Human Subjects

The study protocol conformed to the ethical guidelines of the 1975 Declaration of Helsinki as reflected in a priori approval by the Ethics Committees of the First Affiliated Hospital of Keimyung University Dongsan Medical Center (NO. DSMC 2022–03-011), and written informed consent was obtained from all participants. Human serum and liver tissues from patients with NASH at various stages were obtained from Keimyung University Dongsan Medical Center Biobank. Based on the patient dataset provided by human biobank at Dongsan Medical Center, the average and median ranges for normal patients (n = 36) and MASH patients (n = 35) to represent the physical and biochemical parameters have been previously described^16^.

### qRT-PCR

For liver tissues, primary hepatocytes, and HepG2 cell lines, RNA was isolated by using TRIzol reagent (15596018, Life Technologies). 1 ~ 3 μg of RNA was used for reverse-transcription PCR to generate cDNA with ReverTra Ace qPCR RT kit (FSQ-101, Toyobo). The expression levels of mRNA were detected using the CFX96TM Real-Time System (Bio-Rad Laboratories). qRT-PCR weas conducted using SYBR Green Master Mix (DQ384–40h, Biofact). *Cyclophilin* or *Tbp* was used as endogenous control gene. Primer sequences are listed in supplemental table 1.

### Histology

Liver tissue was harvested and fixed in 4% paraformaldehyde (Biosesang, P2031). Paraffin-embedding and sectioning for H&E staining was completed at the Woodang network in South Korea.

### Fatty acid oxidation assay

Fatty acid oxidation assay was performed as described previously^11^. Primary hepatocytes were incubated in serum-free William E medium for 16 h and then treated with fatty acid oxidation media (0.3% fatty acid free BSA, 100 μM palmitate (Sigma-Aldrich, P9767), 0.4 μCi [1-^14^C] palmitate (Perkin Elmer, NEC075H050UC), 1mM carnitine (Sigma Aldrich, 8.40092) in William’s medium E media in 24-well plates for 3 hours at 37°C. 400 μl of the media was added to acidification vials, which have filter paper soaked with 40 μl of aM NaOH under the cap and 200 μl of 1 M perchloric acid in the tube. After 1 hour, captured ^14^CO_2_ and acid-soluble metabolites (ASM) were used to measure radioactivity for fatty acid oxidation rates, and the CO_2_/ASM ratio was calculated for complete oxidation rate. The cells on the plates were lysed with 0.1 M HCl to quantify protein concentrations.

### Measurement of mitochondrial DNA (mtDNA) copy number

Mitochondrial DNA copy number was analyzed as described in previous studies^17^. Same methods were used for HepG2 cell lines and liver tissues. 20 mg of liver tissue was added to 600 μl of lysis buffer (0.2 mg/ml Proteinase K (Roche, 03115836001), 100 mM NaCl, 10 mM EDTA, 0.5% SDS, 20 mM Tris-HCl (pH7.4)) and incubated overnight at 55 °C. 100 μg/ml of RNase A (Roche Diagnostics, 10109169001) was added and samples were incubated at 37 °C for 30 min. Samples were mixed with 250 μl of 7.5 M ammonium acetate and 600 μl of isopropyl alcohol and then centrifuged at 15000 × g for 10 min at 4 °C. Pellets were washed with 70% ethanol, dried, and solubilized in TE buffer. qRT-PCR was used to measure relative numbers of copies of mtDNA and nuclear DNA. Mitochondrial DNA quantities were analyzed with mitochondrial DNA specific sequences and normalized with nuclear DNA sequences (GAPDH or HK2). Primer sequences are listed in supplemental table 2.

### Mitochondria fractionation for immunoblot analysis

Mitochondria was isolated as described in previous studies ^18^. Briefly, cells were minced in mitochondrial isolation buffer (MSHE, 70 mM sucrose, 210 mM mannitol, 5 mM HEPES, 1mM EGTA, pH 7.2) by stroking 5 times with a Teflon glass homogenizer. The homogenate was centrifuged at 800 × g for 10 min and the supernatant was centrifuged at 8000 × g for 10 min to pellet the mitochondrial fraction. The pellet was completely resuspended with MSHE buffer and was centrifuged at 8000 × g to wash mitochondria pellet. Mitochondria pellet was resuspended with RIPA buffer to isolate protein samples. All steps for mitochondria isolation were performed on ice.

### Site-directed mutagenesis

P62 S403A and p62 S403E overexpression plasmids were generated by using CloneAmp Hifi DNA PCR premix (Takara, 639298) with 0.1 μg p62 WT plasmid. PCR products were treated with DpnI (NEB) to remove circular double stranded templates from the reaction. After agarose gel purification, eluted DNA was ligated by using In-Fusion HD enzyme premix (Takara, 639690) and transformed into Stellar Competent Cells (Clontech, 636763).

### Transfection

Mouse primary hepatocytes were transfected with siRNA (5 pmol per well of 24 well plate) against negative control or TBK1 by using Lipofectamine RNAiMAX (Life Technologies, 13778500). HepG2 cells were transfected with a combination of pcDNA-flag-human TBK1 WT, pcDNA-flag-human TBK1 K^38^A plasmids, as described in each experiment, using Lipofectamine 3000 (Life Technologies). Primary hepatocytes were transfected with pcDNA-flag-human TBK1 WT or pcDNA-flag-human TBK1 K^38^A by using Lipofectamine LTX (Life Technologies, A12621). Cells were harvested after 48–72 hours of transfection for further assays. For mitoKeima assay, mtKeima plasmid was overexpressed in TBK1 WT or TBK1 KO via Lipofectamine 3000.

### Immunoblot analysis

Cells or liver tissues were lysed in RIPA buffer (25 mM Tris/HCl pH7.6, 150 mM NaCl, 1% NP-40, 1% sodium deoxycholate, 0.1% SDS) with 1 mM DTT and protease and phosphatase inhibitor cocktail (Thermo Fisher Scientific). After centrifuging the lysates at 14000 × g for 15 min at 4°C, the supernatant was used for protein quantification using BCA protein assay kits (Pierce, 23227). Primary antibodies were used at a 1:1,000 dilution unless otherwise specified and purchased from Cell Signaling: pS172 TBK1 (5483S), TBK1 (3031S), b-actin (4970), cleaved caspase 3 (9661), pY705 STAT3 (9131), STAT3 (9139), pT108/Y182 p38 (9211), p38 (9212), ATG12 (4180), ATG5 (12994), ATG7 (8558), pS403 p62 (39786), p62 (5114), TOM20 (42406), LC3 (12741), pS2481 mTOR (2974), mTOR (2983), pT389 S6K (9205), S6K (9202), or Abcam: OXPHOS (ab110413), or Abclonal: LAMP1 (A2582).

### Fluorescence dye staining

500 nM of MitoTracker-DeepRed (Invitrogen, M46753) or 200 nM of Mitotracker-Green (Invitrogen, M46750) for mitochondria staining and 1 μM of Lysotracker green (Invitrogen, L7526) for lysosome activity analysis were incubated for 30 minutes before harvesting cells. After staining, cells were analyzed via confocal microscope or flow cytometry. 10 μM of DQ-Red (Invitrogen, D12051) was incubated for 4 hours.

### Mitochondrial quality measurement

JC-1 dye (abcam, ab141387) or TMRE dye (Invitrogen, T669) were used to analyze mitochondria membrane potential. Briefly, cells were incubated with 5 μg/ml JC-1 dye at 37°C for 1h, and analyzed using FACS CantoII (BD Bioscience) or Cytoflex SRT (Beckman Coulter). For flow cytometry analysis, gates were set with unstained and CCCP-treated cells and quantified emission filters appropriate for PE and FITC. Depolarized mitochondria ratio was calculated as FITC/PE ratio. For mitochondrial ROS measurement, 2 μM of mitoSOX (Invitrogen, M36009) was incubated for 30 minutes, and then analyzed by FACS CantoII.

### Quantification and Statistical analysis

Statistical analyses were performed with Prism software version 10.4.0 (GraphPad Software). Comparisons of two groups were made by conducting Student’s *t*-tests. For more than two groups, we evaluated the data with one-way ANOVA or two-way ANOVA (Holm-Sidak’s multiple comparisons) test. Differences were considered statistically significant if p < 0.05 (* or #); p < 0.01 (** or ##) or p < 0.001 (*** or ###). The statistical methods of each experiment are indicated in the figure legends.

## RESULTS

### TBK1 is associated with maintenance of mitochondrial quality control

**Figure 1.**
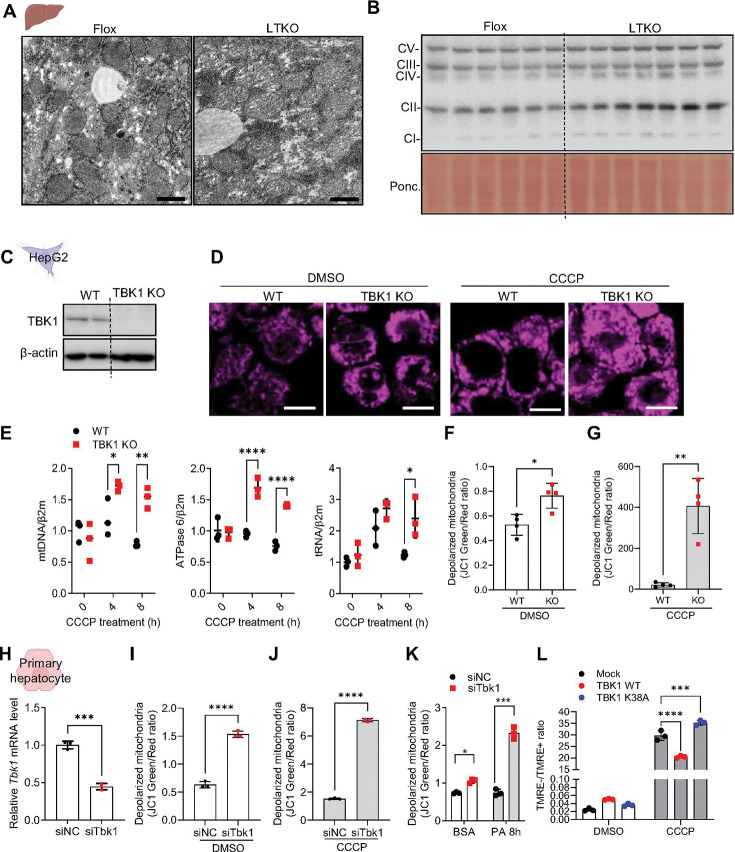
TBK1 is associated with maintenance of mitochondrial quality control. (A) Transmission electron microscopy (TEM) images of liver sections from HFD-fed flox and liver-specific TBK1 knockout (LTKO) mice. Scale bar, 1 μm. (B) Immunoblot analysis of OXPHOS complex proteins in liver tissues from HFD-fed flox and LTKO mice (n = 6 or 7). (C) Immunoblot of TBK1 protein levels in CRISPR-Cas9–mediated TBK1 knockout (KO) HepG2 cells compared to wild-type (WT) HepG2 cells (n = 6). (D) Representative images of MitoTracker Deep Red staining in WT or TBK1 KO HepG2 cells treated with DMSO or 20 mM of CCCP for 4 h. (E) Mitochondrial DNA (mtDNA) copy number in WT and TBK1 KO HepG2 cells following 20 mM of CCCP treatment, measured using mtDNA-specific primers (mtDNA, ATPase6, tRNA). Nuclear DNA (β2-microglobulin) was used for normalization (n = 3). (F,G) Quantification of mitochondrial depolarization under basal conditions(F) and CCCP treatment for 2 hours (G) using JC-1 green/red fluorescence ratio in WT and TBK1 KO HepG2 cells (n = 4). (H) *Tbk1* mRNA expression levels in mouse primary hepatocytes transfected with control siRNA (siNC) or *Tbk1*-targeting siRNA (siTbk1) (n = 3). (I, J) Mitochondrial depolarization in siNC or siTbk1-transfected primary hepatocytes under basal (I) or CCCP-treated (J) conditions (n = 3). (K) Mitochondrial depolarization in *Tbk1*knockdown hepatocytes treated with BSA or 200 μM palmitic acid for 8 h (n = 3). (L) Mitochondrial depolarization measured by the ratio of TMRE-negative to TMRE-positive cells in primary hepatocytes overexpressing either WT TBK1 or kinase-dead TBK1 (K38A) (n = 3). * *p* < 0.05, **p < 0.01, ***p < 0.001, ***p < 0.0001. Data are presented as mean ± SD. Statistical significance was determined by unpaired two-tailed Student’s *t*-test (F–J) or two-way ANOVA (E, K, L).

To investigate the role of TBK1 in hepatic function, we assessed the quantitative and qualitative changes in mitochondria induced by TBK1 deficiency in hepatocytes. We conducted a series of experiments using liver tissue from liver (hepatocyte)-specific TBK1 KO (LTKO) mice. Electron microscopy revealed that the mitochondria in the liver of LTKO mice appeared swollen, indicating morphological alterations associated with TBK1 deficiency (Fig. 1A) as shown in our previous study ^11^. This alteration in mitochondria was further supported by a quantitative analysis of OXPHOS complex proteins, which showed a significant increase in their expression in LTKO liver tissue, consistent with previous reports (Fig. 1B) ^11^.

To explore further the impact of TBK1 deficiency on mitochondrial quantity, we generated a TBK1-deficient HepG2 cell line using the CRISPR-Cas9 system. TBK1 knockout was confirmed, and subsequent staining with Mitotracker revealed a significant increase in mitochondrial mass in TBK1-deficient cells (Fig. 1C, D). Notably, when subjected to mitochondrial depolarization induced by CCCP, TBK1-deficient HepG2 cells exhibited an increase in mitochondrial DNA (mtDNA) copy number, indicating a rise in mitochondrial quantity compared to control cells, which showed no significant change (Fig. 1E). However, mitochondrial biogenesis-related gene expression was not altered in TBK1 KO HepG2 cells (Supplementary Fig. 1A). These findings collectively suggest that TBK1 deficiency leads to an increase in mitochondrial quantity in hepatocytes without altering biogenesis.

In parallel with the observed increase in mitochondrial quantity, we assessed mitochondrial quality by measuring mitochondrial depolarization using JC1 dye staining. TBK1-deficient HepG2 cells showed a higher proportion of depolarized mitochondria under basal conditions, and this effect was more pronounced following CCCP treatment (Fig. 1F, G).

To assess whether these findings extend to normal hepatocytes, we silenced TBK1 expression in mouse primary hepatocytes using siRNA. Consistent with observations in TBK1-deficient HepG2 cells, TBK1 knockdown led to increased mitochondrial depolarization both under basal conditions and following CCCP treatment (Fig. 1H-J) without significant alterations in the expression of genes related to mitochondrial biogenesis (Supplementary Fig. 1B). Given that hepatic mitochondria are exposed to elevated fatty acid levels in MASLD, we next treated TBK1-deficient cells with 200 μM palmitic acid. This led to a further increase in mitochondrial depolarization in the absence of CCCP (Fig. 1K). Moreover, overexpression of wild-type TBK1 ameliorated CCCP-induced mitochondrial depolarization, whereas expression of the kinase-dead mutant (K38A) had no such effect (Fig. 1L). Collectively, these findings indicate that TBK1 deficiency results in the accumulation of dysfunctional mitochondria, underscoring its critical role in maintaining mitochondrial quality control in hepatocytes.

### TBK1 deficient hepatocytes exhibit increased susceptibility to mitochondrial stress

**Figure 2.**
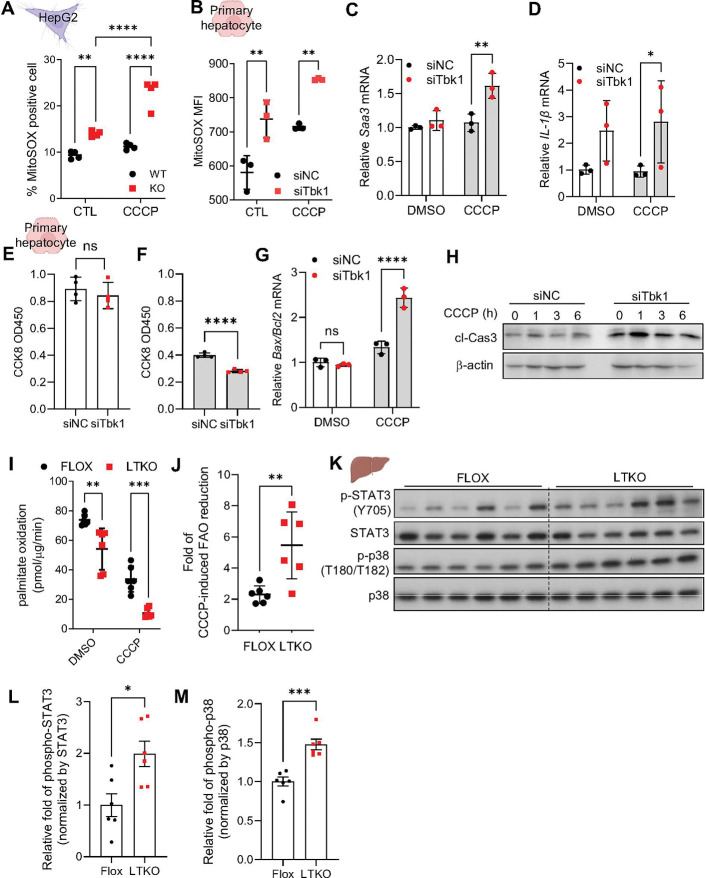
TBK1-deficient hepatocytes exhibit increased susceptibility to mitochondrial stress. (A) Mitochondrial ROS levels measured by MitoSOX staining in WT and TBK1 KO HepG2 cells (n = 4). (B) Mitochondrial ROS levels in primary hepatocytes transfected with siNC or siTbk1 (n = 3). (C, D) mRNA expression of proinflammatory genes *Saa3* (C) and *Il1b* (D) in siNC or siTbk1-transfected primary hepatocytes (n = 3). (E, F) Cell viability measured by CCK-8 assay in primary hepatocytes under basal conditions (E) and following 10 mM of CCCP treatment (F) (n = 3). (G) *Bax/Bcl2* mRNA expression ratio in primary hepatocytes under CCCP-induced stress (n = 3). (H) Immunoblot of cleaved caspase-3 in primary hepatocytes treated with CCCP. (I) Basal ^14^C-palmitate oxidation activity in primary hepatocytes from flox and LTKO mice (n = 6). (J) Fold change in CCCP-induced suppression of palmitate oxidation activity in flox versus LTKO hepatocytes (n = 6). (K) Immunoblot analysis of phosphorylated and total STAT3 and p38 in liver tissues from flox and LTKO mice (n = 6). (L,M) Quantification of phospho-STAT3 (L) and phospho-p38 (M), normalized to their respective total protein levels (n = 6). * *p* < 0.05, **p < 0.01, ***p < 0.001, ***p < 0.0001. Data are presented as mean ± SD (A–G, I, J) or mean ± SEM (L, M). Statistical significance was assessed by unpaired two-tailed Student’s t-test (E, F, J, L, M) or two-way ANOVA (A–D, G, I).

To investigate the impact of TBK1 deficiency on hepatocyte fitness through the accumulation of depolarized mitochondria, we first measured mitochondrial reactive oxygen species (ROS). We observed a significant increase in mitochondrial ROS, as indicated by a mitoSOX signal^19^, in TBK1-deficient compared to wild-type (WT) HepG2 cells, indicating elevated mitochondrial ROS levels (Fig. 2A). Moreover, TBK1-deficient cells exhibited heightened sensitivity to mitochondrial ROS induction upon CCCP treatment, showing a more pronounced increase in ROS compared to WT cells. Similar patterns were observed in mouse primary hepatocytes with reduced TBK1 expression via siRNA, further confirming these findings (Fig. 2B). In addition, gene expression levels of inflammatory genes such as *Saa3* and *Il1b* were significantly enhanced in TBK1 deficient hepatocytes (Fig. 2C, D), possibly reflecting increased mitochondrial damage, although we note that TBK1 activity can inhibit noncanonical NFκB activation via phosphorylation and degradation of NIK in adipocyte^13^. In parallel, we assessed cell viability under these conditions using the CCK-8 assay. We found that inhibition of TBK1 expression significantly increased CCCP-induced cell death (Fig. 2E, F). Additionally, the ratio of *Bax*/*Bcl-2* transcripts and cleaved caspase-3 levels were elevated in the *Tbk1* siRNA cells, suggesting that TBK1 may play a crucial role in maintaining cell viability under mitochondrial stress conditions (Fig. 2G, H). Furthermore, given the essential role of mitochondria in fatty acid oxidation (FAO), we measured FAO activity and found that basal FAO was reduced in TBK1 knockout (KO) cells (Fig. 2I), consistent with previous reports^11^. Upon CCCP treatment, FAO was significantly decreased in TBK1-deficient compared to WT cells, indicating a greater impairment of mitochondrial function in the absence of TBK1 (Fig. 2J). Consistent with upregulated mitochondrial ROS and stress responses in TBK1 KO hepatocytes in cell culture, LTKO mice also showed significantly increased phosphorylation of STAT3 and p38 in liver (Fig. 2K-M). These data suggest that TBK1 deficiency increases sensitivity to mitochondrial stress, leading to impaired mitochondrial function, such as reduced fatty acid oxidation, and heightened susceptibility to cell death as well as inflammation.

### TBK1 deficiency impairs the clearance of depolarized mitochondria in the liver

**Figure 3.**
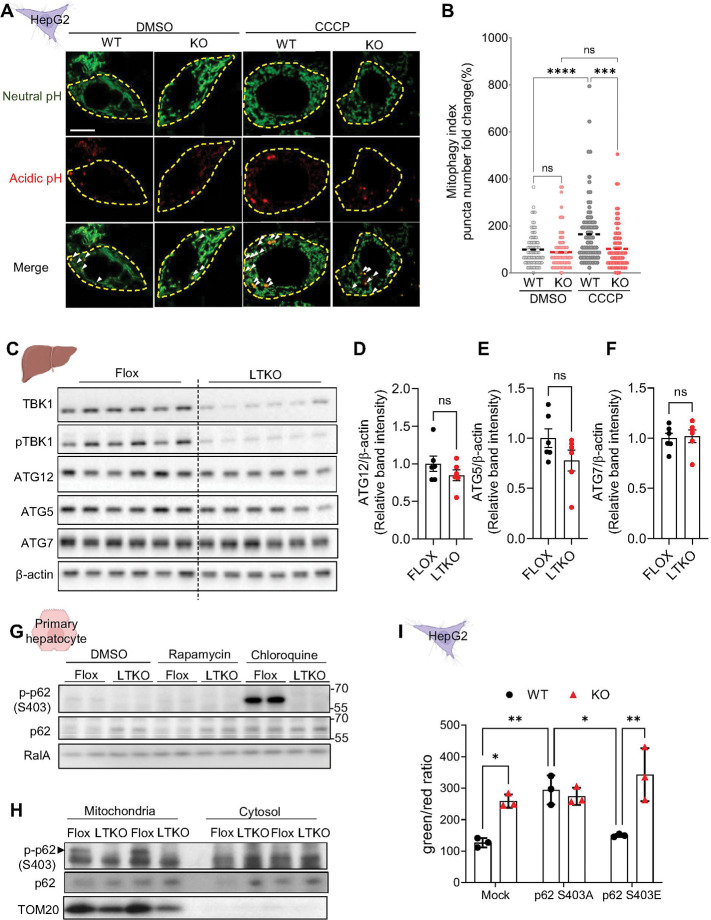
TBK1 deficiency impairs mitophagy, but the effect is not rescued by p62 phosphorylation. (A) Representative images of mtKeima-based mitophagy assay in WT and TBK1 KO HepG2 cells under 20 mM of CCCP treatment for 4 hours. (B) Quantification of red puncta per cell representing mitolysosomes from image (A). (C) Immunoblot analysis of autophagy-related proteins ATG12, ATG5, and ATG7 in liver tissues from flox and LTKO mice (n = 6). (D–F) Quantification of ATG12 (D), ATG5 (E), and ATG7 (F) protein levels normalized to β-actin. (G) Immunoblot analysis of phosphorylated p62 (Ser403) and total p62 in primary hepatocytes from flox and LTKO mice. (H) Immunoblot analysis of phospho-p62 (Ser403) and total p62 in crude mitochondrial and cytosolic fractions of primary hepatocytes from flox and LTKO mice. </p/>(I) Quantification of mitochondrial depolarization using JC-1 dye in TBK1 KO HepG2 cells overexpressing p62 S403A or p62 S403E mutants (n = 3). * *p* < 0.05, **p < 0.01, ***p < 0.001, ***p < 0.0001. Data are presented as mean ± SD (B, I) or mean ± SEM (D–F). Statistical significance was determined using unpaired two-tailed Student’s t-test (D–F) or two-way ANOVA (B, I).

To investigate the mechanism underlying the increase in depolarized mitochondria observed in TBK1-deficient hepatocytes, we examined whether this was due to impaired clearance of depolarized mitochondria. A Mito-Keima assay was used to detect mitochondrial localization to lysosomes, which leads to degradation of damaged mitochondria. TBK1 KO HepG2 cells showed impaired mitochondrial localization into the lysosomal fraction upon CCCP treatment (Fig. 3A, B). Analysis of liver tissue from LTKO mice revealed no significant differences in protein expression levels of genes associated with general autophagy between genotypes (Fig. 3C-F). These findings imply that the impaired mitophagy in TBK1-deficient hepatocytes may not be attributed to altered abundance of core autophagy-related proteins such as ATG12, ATG5, and ATG7, suggesting a more specific disruption in mitophagy regulation rather than general autophagy machinery.

### TBK1 is necessary for p62 phosphorylation, but is not sufficient for mitophagy restoration in TBK1 deficient hepatocytes

To investigate the mechanism by which TBK1 regulates mitophagy in hepatocytes, we compared the recruitment of autophagy adaptor proteins, such as p62, which are known to be regulated by TBK1 in other cell types^20^. Primary hepatocytes were treated with rapamycin to induce autophagy. Phosphorylated p62 was barely detectable under these conditions. In contrast, treatment with chloroquine (CQ), which inhibits lysosomal degradation, led to the accumulation of phosphorylated p62 in wild-type cells, but not in TBK1 KO cells (Fig. 3G), indicating that TBK1 is essential for p62 Ser403 phosphorylation during autophagic flux. Consistent with impaired mitophagy, p62 protein levels were elevated in TBK1-deficient hepatocytes under basal conditions, while this difference was abolished upon CQ treatment, suggesting a defect in p62 turnover. Furthermore, phosphorylated p62 was preferentially enriched in the mitochondrial compared to the cytosolic fraction, a pattern that was lost in TBK1 KO cells (Fig. 3H), supporting a role for TBK1 in facilitating p62-mediated cargo recognition and mitochondrial targeting during mitophagy. To examine the potential role of p62 phosphorylation in mitophagy inhibition in TBK1-deficient cells, we overexpressed phospho-defective mutant p62 (S403A), and a phospho-mimetic mutant p62 (S403E) in TBK1 KO HepG2 cells and compared the level of mitochondrial depolarization. Notably, mitophagy was not restored by overexpression of the phospho-mimetic mutant p62 (Fig. 3I). This suggests that while p62 S403E-mediated p62 activation enhances cargo delivery to lysosomes, its clearance might be impaired in the absence of TBK1. These findings indicate that p62 phosphorylation alone is insufficient to rescue mitophagy in TBK1-deficient cells.

### TBK1 deficiency shows defective lysosome activity

**Figure 4.**
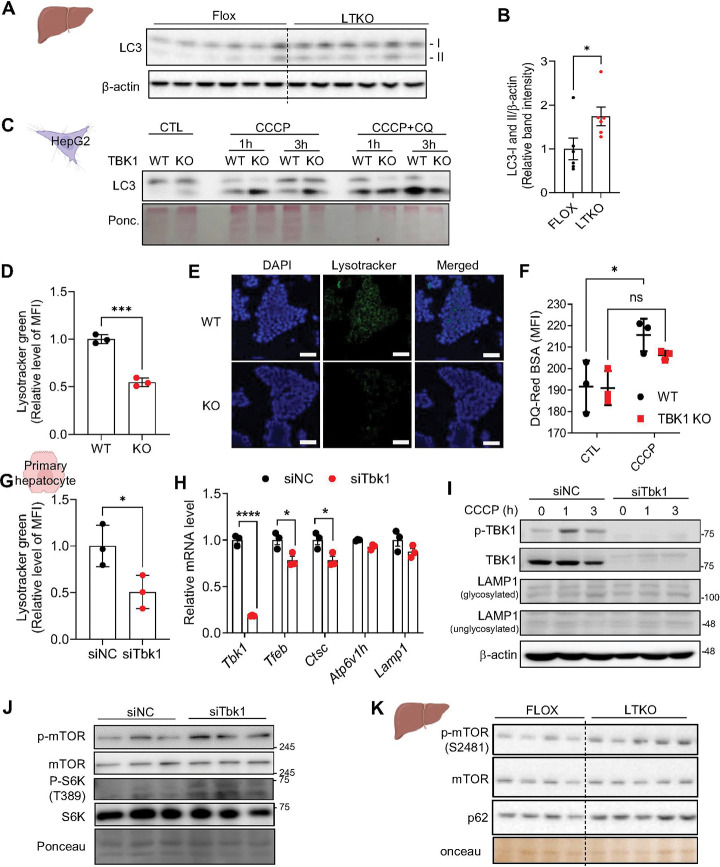
TBK1-deficiency shows defective lysosomal activity. (A) Immunoblot analysis of LC3-I and LC3-II levels in liver tissues from HFD-fed flox and LTKO mice (n = 6). (B) Quantification of LC3-I and LC3-II levels normalized to β-actin (n = 6). (C) Immunoblot of LC3-I and LC3-II in WT and TBK1 KO HepG2 cells treated with CCCP or CCCP in combination with chloroquine (CQ) for 6 hours. (D) Mean fluorescence intensity (MFI) of LysoTracker Green in WT and TBK1 KO HepG2 cells (n = 3). (E) Representative confocal microscopy images of LysoTracker Green staining in WT and TBK1 KO HepG2 cells. Scale bar indicates 50 μm. (F) Lysosomal proteolytic activity assessed by DQ-Red BSA fluorescence in WT and TBK1 KO HepG2 cells (n = 3). (G) MFI of LysoTracker Green in siNC- and siTbk1-transfected primary hepatocytes (n = 3). (H) Relative mRNA expression levels of lysosomal biogenesis-associated genes in siNC- and siTbk1-transfected primary hepatocytes (n = 3). (I) Immunoblot of phosphorylated TBK1 (p-TBK1), total TBK1, and LAMP1 in siNC- and siTbk1-transfected primary hepatocytes upon CCCP treatment. (J) Immunoblot analysis of phosphorylated mTOR and S6K levels in siNC- and siTbk1-transfected primary hepatocytes treated with 10μM CCCP for 6 hours. (K) Immunoblot analysis of phosphorylated mTOR and mTOR levels in liver from NCD-fed flox versus LTKO mice (n = 4~5). * *p* < 0.05, **p < 0.01, ns. no significance; *p*> 0.05. Data are presented as mean ± SD (D, F, G, I) or mean ± SEM (B). Statistical significance was assessed using unpaired two-tailed Student’s t-test (B, D, G, H) or two-way ANOVA (F).

Another notable phenotype observed in LTKO mice was a marked increase in both LC3-I and LC3-II levels in the liver (Fig. 4A, B), suggestive of altered autophagic flux. Further investigation revealed elevated LC3-II accumulation in the mitochondrial fraction of TBK1 KO HepG2 cells following CCCP treatment. Interestingly, this difference was abolished upon co-treatment with chloroquine (CQ), a lysosomal inhibitor, implicating defective lysosomal degradation as a key contributor to impaired mitophagy in TBK1-deficient cells (Fig. 4C). Moreover, combined CCCP and CQ treatment resulted in an overall reduction in total LC3 levels in the TBK1 KO group, suggesting that TBK1 deficiency not only disrupts lysosomal clearance but may also compromise the initiation of mitophagy. Collectively, these findings indicate that TBK1 is essential for effective lysosomal degradation of damaged mitochondria, and its loss leads to mitophagy impairment and the accumulation of dysfunctional mitochondria.

### Suppressed lysosomal activity is involved in impaired mitophagy in TBK1 deficiency

Given the observed inhibition of lysosomal degradation indicated by LC3 accumulation, we further investigated the changes in lysosomal activity caused by TBK1 deficiency. Lysotracker staining showed reduced intensity in both TBK1 KO HepG2 cells and TBK1 knockdown primary hepatocytes, suggesting a decrease in basal lysosomal activity (Fig. 4D, E). Furthermore, induction of fluorescence intensity of DQ-Red BSA, used to assess lysosomal enzyme activity, was decreased in TBK1 KO cells following CCCP treatment (Fig. 4F). This reduced lysosomal activity in TBK1 deficiency was consistently observed in TBK1 knockdown primary hepatocytes (Fig. 4G).

To further assess the role of TBK1 in lysosomal biogenesis, we examined the mRNA levels of lysosome biogenesis-related gene expression in siTbk1 hepatocytes. Interestingly, knockdown of TBK1 led to a slight reduction of lysosomal markers, including transcription factor EB (*Tfeb*), cathepsin C (*Ctsc*), and *Atp6v1h*, while lysosomal-associated membrane protein 1 (LAMP1), a key lysosomal membrane protein, levels remained unaffected (Fig. 4H, I).

To explore further the mechanism of defective lysosomal activity in TBK1 deficiency, we analyzed the activation of the mammalian target of rapamycin (mTOR) pathway, a known suppressor of lysosomal function. In TBK1-deficient cells, basal phosphorylation of mTOR and of S6K was elevated, indicating a potential inhibitory role of TBK1 on mTOR activity (Fig. 4J). Consistently, liver tissues from LTKO mice also showed increased mTOR phosphorylation, in alignment with *in vitro* observations (Fig. 4K). These findings collectively indicate that TBK1 contributes to the maintenance of lysosomal activity, potentially by suppressing mTOR signaling to support both lysosomal biogenesis and degradative capacity.

### TBK1 activity is reduced in MASH mouse model

**Figure 5.**
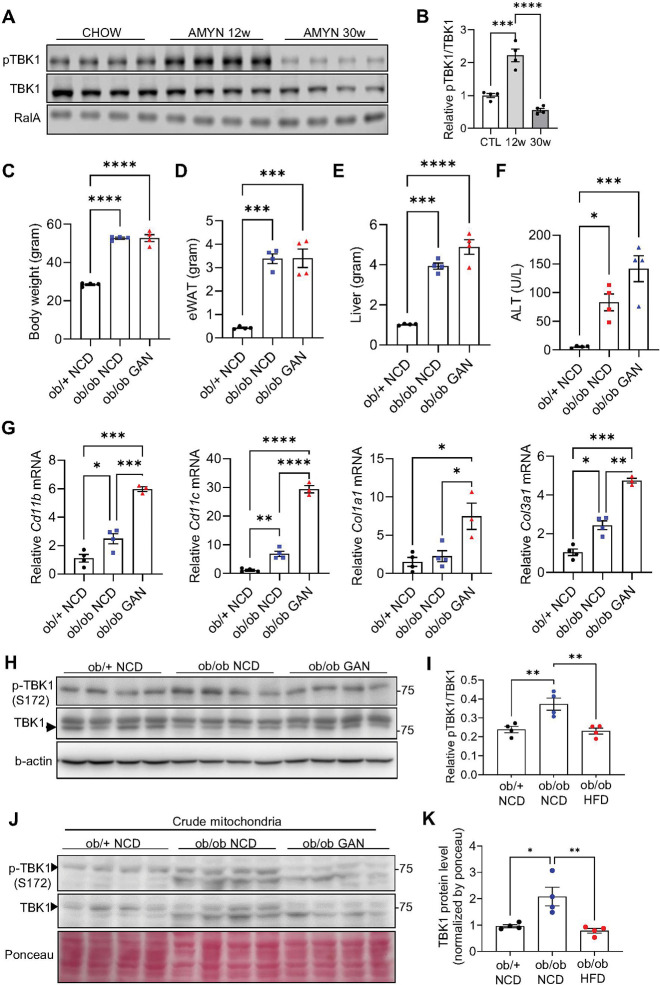
TBK1 kinase activity is reduced in MASH liver. (A) Immunoblot analysis of phosphorylated TBK1 (p-TBK1) and total TBK1 in liver tissues from chow- or Amylin diet-fed mice (n = 4). (B) Quantification of p-TBK1 levels normalized to total TBK1 in mitochondrial fractions (n = 4). (C) Body weight of NCD-fed *ob/+* (lean), NCD-fed *ob/ob* (steatosis), and GAN diet-fed *ob/ob* (MASH) mice (n = 4). (D) eWAT weight across the same three groups (n = 4). (E) Liver weight across the same three groups (n = 4). (F) Serum ALT levels in the three groups (n = 4). (G) mRNA expression levels of inflammatory and fibrosis-related genes in liver tissues from the three groups (n = 4). (H) Immunoblot analysis of p-TBK1 and total TBK1 in liver lysates from NCD-fed *ob/+*, NCD-fed *ob/ob*, and GAN-fed *ob/ob* mice (n = 4). (I) Quantification of p-TBK1 levels normalized to total TBK1 (n = 4). (J) Immunoblot of p-TBK1 and total TBK1 in crude mitochondrial fractions from NCD-fed *ob/+* (lean), NCD-fed *ob/ob*(steatosis), and GAN diet-fed *ob/ob*(MASH) mice (n = 4). (K) Quantification of mitochondrial TBK1 protein level (n = 4). * *p* < 0.05, **p < 0.01, ***p < 0.001, ***p < 0.0001. Data are presented as mean ± SEM. Statistical significance was determined using one-way ANOVA.

To investigate whether TBK1-mediated mitophagy might ameliorate MASH pathogenesis, we first assessed TBK1 expression and activation in liver tissues from MASLD mouse models. While TBK1 phosphorylation was markedly elevated after 12 weeks of amylin diet, consistent with previous reports of increased activity after high fat feeding^11^, long-term amylin diet feeding (30 weeks), which induces MASH-like pathology, including fibrosis and hepatocellular death, resulted in a marked reduction in TBK1 phosphorylation, indicating impaired TBK1 activity (Fig. 5A, B). We generated a second MASLD mouse model, in which *ob/ob* mice were fed either a normal chow diet (NCD) or a high-fat, high-fructose, high-cholesterol (Gubra-Amylin NASH, GAN) diet for 4 weeks to mimic steatosis or MASH stages, respectively. Compared to the lean control group (*ob/+*), both *ob/ob* NCD and *ob/ob* GAN diet groups had higher body weights, but no significant difference was observed between the *ob/ob* NCD and *ob/ob* GAN groups (Fig. 5C). Similar patterns were noted for the mass of eWAT and liver, and ALT levels (Fig. 5D-F). Inflammatory markers, *Cd11b* and *Cd11c*, were significantly higher in the MASH stage, along with increased collagen transcript levels, indicating that the three groups successfully mimic healthy, steatosis, and MASH stages (Fig. 5G).

The kinase activity of TBK1 was significantly reduced in *ob/ob* GAN diet-fed group compared with *ob/ob* NCD-fed group (Fig. 5H, I). In addition, public single cell RNAseq data from the Liver Cell Atlas^21^ revealed decreased *Tbk1* mRNA expression in specific hepatocyte populations from MASLD mice (Supplementary Fig. 2). The protein levels of phosphorylated TBK1 associated with mitochondria are reduced in *ob/ob* GAN diet compared to *ob/ob* NCD-fed mice (Fig. 5J, K). Collectively, these results demonstrate that TBK1 kinase activity is progressively diminished during the transition from steatosis to MASH, particularly within hepatocyte mitochondria, highlighting a potential mechanistic link between TBK1 dysfunction and the exacerbation of MASH pathogenesis.

### TBK1 overexpression can ameliorate MASH development

**Figure 6.**
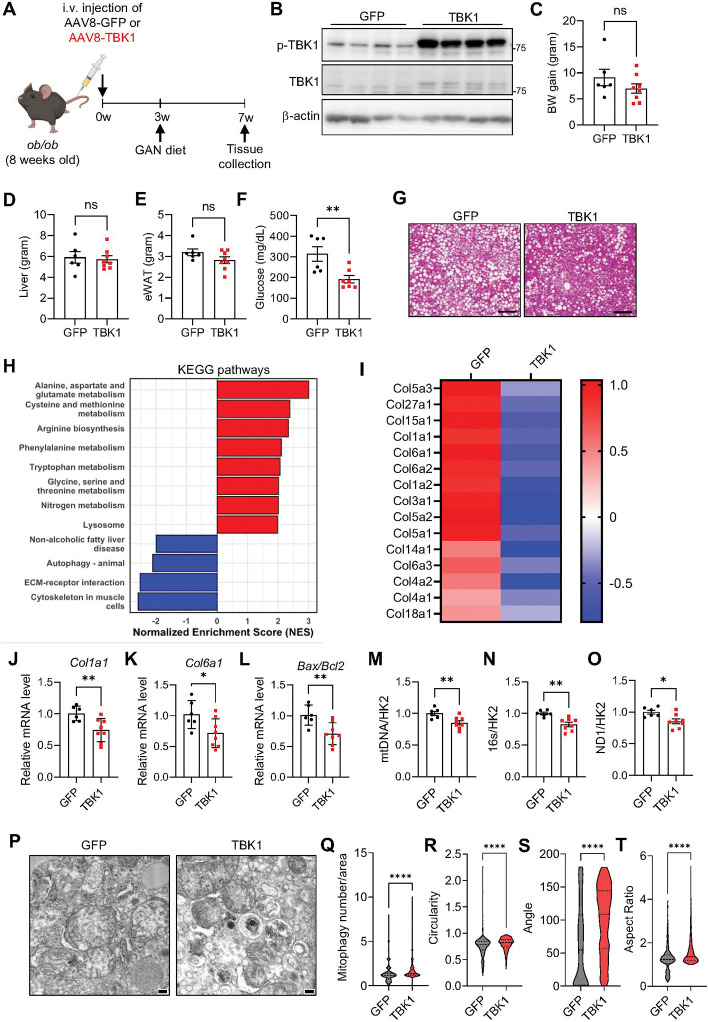
TBK1 overexpression ameliorates MASH progression in vivo. (A) Experimental scheme for intravenous administration of AAV8-TBK1 in the MASH mouse model. (B) Immunoblot analysis of phosphorylated TBK1 (p-TBK1) and total TBK1 in liver tissues following AAV8-TBK1 overexpression (n = 4). (C–E) Body weight gain (C), liver weight (D), and epididymal white adipose tissue (eWAT) weight (E) in AAV8-GFP and AAV8-TBK1 groups (n = 6). (F) Fasting blood glucose levels (n = 6). (G) Representative H&E-stained liver histological images from each group. Scale bar, 200 μm. (H) KEGG pathway enrichment analysis of RNA-seq data from liver tissues of each group. (I) Relative mRNA expression of fibrosis-related collagen genes in liver tissues (n = 3). (J–L) qPCR analysis of *Col1a1* (J), *Col6a1* (K), and *Bax/Bcl2*ratio (L) in liver tissues (n = 6). (M,N,O) Mitochondrial DNA copy number normalized to hexokinase-2 (HK2) in liver tissues (n = 6). (P) Transmission electron microscopy (TEM) images of liver tissues. Scale bar, 200 nm. (Q-T) Quantification of mitochondrial morphology by TEM: mitophagy-associated vesicular mitochondria per area (Q), mitochondrial circularity (R), angle (S), and aspect ratio (T) (n = 6). * *p* < 0.05, **p < 0.01, ***p < 0.001, ***p < 0.0001, ns. no significance; *p* > 0.05. Data are presented as mean ± SEM. Statistical significance was determined using unpaired two-tailed Student’s t-test.

To determine if MASH progression can be ameliorated by overexpressing TBK1, AAV8-TBK1 was administered intravenously to *ob/ob* mice, followed by a MASH-inducing GAN diet for 4 weeks (Fig. 6A). Immunoblot analysis confirmed TBK1 overexpression (Fig. 6B). TBK1 overexpression did not affect body weight, liver weight, or eWAT weight (Fig. 6C-E). However, blood glucose levels significantly decreased in the TBK1 overexpression group compared to the GFP control group (Fig. 6F), likely due to increased fatty acid oxidation unrelated to kinase activity. Liver histology with H&E staining showed a reduction in hepatic lipid accumulation by TBK1 overexpression (Fig. 6G).

RNAseq analysis of liver tissues revealed a significant decrease in extracellular matrix (ECM)-related genes, particularly fibrosis-associated collagens (Fig. 6H, I). qRT-PCR further confirmed reduced expression of fibrosis such as *Col1a1* and *Col6a1* (Fig. 6J, K). In addition, cell death marker (*Bax/Bcl2*) gene expression was downregulated (Fig. 6L). To evaluate if TBK1 overexpression enhances mitophagy, mtDNA copy number was assessed and found to be decreased, suggesting a reduction in mitochondria quantity (Fig. 6M-O). Electron microscopy analysis showed an increase in autophagosome-enclosed mitochondria, indicative of ongoing mitophagy, which was quantified and confirmed to increase with TBK1 overexpression (Fig. 6P, Q). Other mitochondrial morphology characters, which includes reduced circularity, higher angle and aspect ratio, reflect improved mitochondrial quality in TBK1 overexpressed group (Fig. 6R-T). These findings suggest that TBK1 overexpression mitigates MASH pathogenesis by promoting mitophagy and reducing fibrosis.

### TBK1 activity shows a negative correlation with severity of MASLD in patients

**Figure 7.**
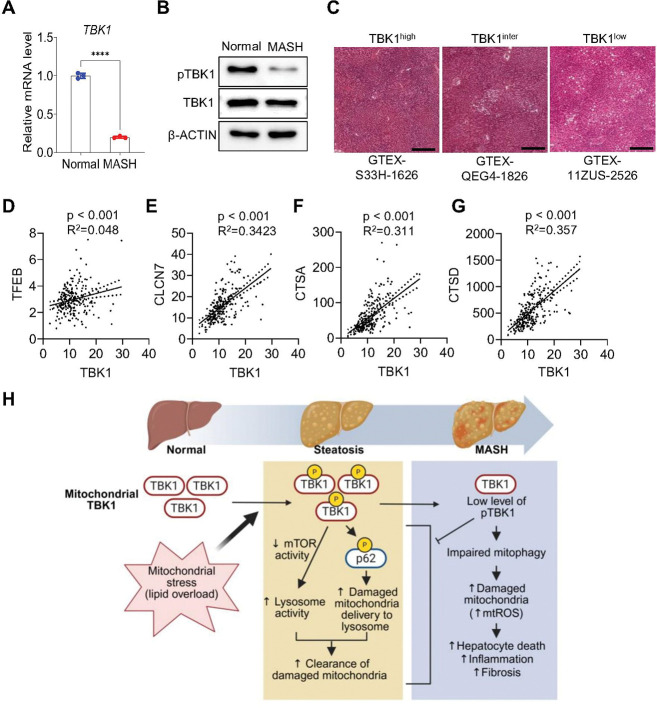
TBK1 activity shows negative correlation with MASLD patients. (A) Relative mRNA expression levels of TBK1 in liver tissues from normal subjects and MASH patients (n = 3 per group). (B) Immunoblot analysis of phosphorylated TBK1 (p-TBK1) and total TBK1 in liver lysates from normal and MASH patient samples. (C) Representative H&E-stained liver histological sections stratified by TBK1 expression levels (high, medium, and low). Scale bars, 400 μm. (D–G) Simple linear regression analysis showing the correlation between *TBK1* mRNA levels and lysosomal biogenesis–associated genes including *TFEB*(D), *CLCN7* (E), *CTSA* (F), and *CTSD* (G) across human liver samples. The data used for the analyses were obtained from: dbGaP accession number phs000424.v8.p2 (H) Proposed model. Upon mitochondrial stress, phosphorylated TBK1 enhances mitochondrial quality control by promoting p62 phosphorylation and lysosomal activity. In MASH liver, however, both the activation and mitochondrial localization of TBK1 are diminished, resulting in impaired mitophagy and the accumulation of damaged mitochondria. This dysfunction exacerbates mitochondrial stress, inflammation, and fibrosis, thereby accelerating MASH progression. ***p < 0.0001. Data are presented as mean ± SEM. Statistical significance was determined by unpaired two-tailed Student’s t-test (A) or linear regression analysis (D–G).

Analysis of human MASH samples showed reduced levels of both total TBK1 and phosphorylated TBK1 (Fig. 7A, B). Increased lipid accumulation in liver was correlated with lower TBK1 expression based on GTEx histology data (Fig. 7C). Furthermore, the mRNA expression level of lysosome-related genes, such as *TFEB*, *CLCN7*, *CTSA*, and *CTSD*, showed a positive correlation with *TBK1* expression in human liver (Fig. 7D-G). These findings highlight the potential role of TBK1 in the regulation of mitophagy and the progression of MASH.

## DISCUSSION

We report here a role for the protein kinase TBK1 in the control of mitochondrial morphology and function. TBK1-deficient hepatocytes and liver tissues from hepatocyte-specific TBK1 knockout (LTKO) mice showed increased mitochondrial number but compromised quality, as indicated by elevated mitochondrial depolarization and ROS levels. Impaired lysosomal activity and reduced mitophagy flux were identified as underlying mechanisms contributing to the accumulation of dysfunctional mitochondria in TBK1-deficient cells. Importantly, TBK1 overexpression improved mitochondrial quality and reduced markers of fibrosis in MASH mouse models, with enhanced mitophagy and decreased level of fibrosis-related gene expression. Our findings also highlight a decrease in TBK1 activity in MASH, observed in both mouse models and human samples, supporting the pathological relevance of TBK1 dysregulation in late stages of MASLD progression (Fig. 7H).

Mitophagy, the selective autophagic degradation of damaged mitochondria, is a vital process for maintaining mitochondrial quality and cellular homeostasis in hepatocytes^22,23^. Previous studies have shown that TBK1 regulates mitophagy by phosphorylating autophagy adaptor proteins such as OPTN, NDP52, and p62, enabling the recruitment of damaged mitochondria to the autophagy machinery^20,24,25^. However, many studies were conducted in non-hepatic cells or artificial settings, such as PENTA knockout systems, which lack multiple cargo receptors including OPTN, CDP52, TAX1BP1, p62, and NBR1 ^9^. Our study builds on these findings by showing that TBK1 is essential for mitophagy in normal hepatocytes, highlighting its physiological relevance in hepatic mitochondrial quality control. Also, we observed p62 Ser403 phosphorylation as a TBK1-dependent mechanism that enhances cargo delivery to lysosomes during hepatic mitophagy. Interestingly, while p62 Ser403 phosphorylation was insufficient to rescue mitophagy in TBK1-deficient cells, it underscores the critical function of TBK1 in coordinating multiple aspects of mitophagy beyond adaptor protein recruitment, including lysosomal function.

Notably, our results highlight the role of TBK1 as a regulator of lysosomal activity in hepatocytes. Lysosomal dysfunction, indicated by reduced lysotracker staining intensity and DQ-BSA fluorescence, was observed in TBK1-deficient cells. Although lysosomal quantity (e.g., LAMP1 levels) was not affected, TBK1 deficiency impaired lysosomal degradation capacity, contributing to the accumulation of damaged mitochondria. This impaired lysosomal activity may provide a mechanistic explanation for previous observations of increased transfection efficiency in TBK1-deficient cells^26^. Given that agents like chloroquine enhance transgene expression by preventing lysosomal DNA degradation, the loss of TBK1 may similarly suppress lysosomal function, thereby stabilizing transfected plasmids and facilitating gene expression.

TBK1-deficient cells also showed hyperactivation of mTOR signaling, which is known to suppress lysosomal biogenesis and activity ^27^. These data suggest that the regulatory role of TBK1 in lysosomal activity may be partially mediated through mTOR modulation. The regulatory relationship between TBK1 and mTOR signaling is complex and appears highly context-dependent, varying with cell type, nutrient status, and disease stage^28–31^. Several studies have reported that TBK1 negatively regulates mTORC1 activity through phosphorylation of Raptor at Ser877, particularly under chronic innate immune activation, such as in Trex1-deficient or prostate cancer models, where TBK1 suppresses mTOR-driven anabolic programs and promotes cellular quiescence or dormancy^28^. Conversely, recent findings suggest that lysosome-associated TBK1 may facilitate mTORC1 activation by relieving Rab7-mediated suppression under amino acid-rich conditions^31^. In our study, TBK1-deficient hepatocytes showed an increase in mTOR signaling, coupled with a marked reduction in lysosomal activity and impaired mitophagy flux. These findings suggest that hepatic TBK1 may play a supportive role in lysosomal maintenance, thereby restraining mTORC1 activity under basal or stressed conditions by sustaining lysosomal degradation capacity.

Our findings suggest a dynamic, stage-dependent regulation of TBK1 kinase activity during the progression of MASLD and MASH. Phosphorylation of TBK1(Ser172), a marker for its kinase activity, is increased during the early stages of hepatic steatosis—likely in response to inflammatory cues. This observation aligns with prior reports indicating TBK1 activation under inflammatory stress ^11^. We propose that during the initial phase of MASLD, TBK1 is phosphorylated as an adaptive response, promoting anabolic pathways via inhibition of AMPK^13^, and facilitating mitophagy to remove damaged mitochondria and maintain mitochondrial homeostasis. However, as the disease advances toward MASH, TBK1 kinase activity becomes markedly diminished despite relatively preserved total protein levels in mice, with reduced TBK1 expression in liver tissue of human MASH patients. This attenuation of TBK1 function may reflect a breakdown of compensatory mitochondrial quality control, thereby contributing to the exacerbation of mitochondrial dysfunction, hepatic inflammation, and fibrosis.

Identifying upstream mechanisms underlying impaired TBK1 activity in the latter stages of MASH remains an important avenue for future research. AMPK is a potential candidate, as it has been shown to phosphorylate TBK1 at Ser511 in response to viral infection, facilitating downstream IRF3 recruitment and innate immune activation ^32^. In support of this, we have also observed that AMPK can indirectly activate TBK1 via ULK1 in adipocytes, suggesting that this axis may serve as a general mechanism linking metabolic stress to TBK1 activation^13^. Given that AMPK activity is diminished in MASH livers ^33^, defective AMPK-TBK1 signaling could contribute to impaired mitophagy and mitochondrial dysfunction. Additionally, the reduction in TBK1 activity may be, at least in part, a secondary effect of hepatocyte injury or loss in advanced stages of disease. Distinguishing primary regulatory defects from downstream consequences of liver damage will be critical to fully understanding TBK1’s role in MASH progression.

We note that numerous studies have demonstrated that treatment with the TBK1/IKKε inhibitor amlexanox ameliorates hepatic steatosis, inflammation, and fibrosis in MASLD models^34–38^. While improvements in body weight, glucose homeostasis, and fat mass have been attributed to TBK1/IKKε inhibition^36,38^, these effects are mediated through multiple organ systems, including adipose tissue, brain and liver. Moreover, amlexanox exhibits pleiotropic actions beyond TBK1 and IKKe, including modulation of GRK5^39,40^, HSP90^41^, S100A13^42^, and FGF1^43^ signaling pathways, although these effects occur at higher doses. A primary effect of amlexanox is to increase energy expenditure in adipocytes via improvement in catecholamine sensitivity, including adipocyte FGF21 and IL-6 induction, which improve metabolic outcomes, suppress hepatic gluconeogenesis and promote glycemic control^36^. These data support the idea that the beneficial effects of amlexanox in MASLD may result primarily from systemic metabolic improvements, rather than direct inhibition of hepatocyte TBK1. Furthermore, amlexanox reduces inflammatory responses in various models^34,35,44–46^. Indeed, amlexanox exhibits cell type–specific effects on immune regulation: it has been reported to promote T cell activation while suppressing macrophage-mediated pro-inflammatory responses^47^. Moreover, amlexanox enhances bile acid synthesis and promotes fecal bile acid secretion in the gut^37^, markedly improved MASH-related dyslipidemia, hepatic steatosis, inflammation, liver injury, and hepatic fibrosis. In addition, amlexanox has shown clear direct effects in the liver, improving dyslipidemia and preventing atherosclerosis^48^. Thus, irrespective of the mechanism, the profound anti-steatotic effects of amlexanox seen in mouse models of MAFLD and MASH are likely to prevent subsequent mitochondrial dysfunction, thus obscuring the potential effects of TBK1 inhibition on mitochondrial quality control.

Collectively, our findings support a model in which TBK1 acts as a metabolic rheostat, integrating inflammatory and nutrient-derived signals to orchestrate mitochondrial quality control and hepatic lipid metabolism, particularly important in the later stages of liver disease. Loss of TBK1 function disrupts this regulatory network, contributing to the accumulation of dysfunctional mitochondria, hepatocellular stress, and fibrosis—hallmarks of MASH pathogenesis. Restoration of TBK1 expression in mouse models improved mitophagy, reduced fibrosis-related gene expression, and ameliorated liver pathology.

## Supplementary Material

Supplementary Files

This is a list of supplementary files associated with this preprint. Click to download.

• 250728TBK1mitophagyfigureSuppleonly.pdf
